# Parameters-tuning of PID controller for automatic voltage regulators using the African buffalo optimization

**DOI:** 10.1371/journal.pone.0175901

**Published:** 2017-04-25

**Authors:** Julius Beneoluchi Odili, Mohd Nizam Mohmad Kahar, A. Noraziah

**Affiliations:** 1 Faculty of Computer Systems and Software Engineering, Universiti Malaysia Pahang, Kuantan, Malaysia; 2 IBM Centre of Excellence, Universiti Malaysia Pahang, Kuantan, Malaysia; Chongqing University, CHINA

## Abstract

In this paper, an attempt is made to apply the African Buffalo Optimization (ABO) to tune the parameters of a PID controller for an effective Automatic Voltage Regulator (AVR). Existing metaheuristic tuning methods have been proven to be quite successful but there were observable areas that need improvements especially in terms of the system’s gain overshoot and steady steady state errors. Using the ABO algorithm where each buffalo location in the herd is a candidate solution to the Proportional-Integral-Derivative parameters was very helpful in addressing these two areas of concern. The encouraging results obtained from the simulation of the PID Controller parameters-tuning using the ABO when compared with the performance of Genetic Algorithm PID (GA-PID), Particle-Swarm Optimization PID (PSO-PID), Ant Colony Optimization PID (ACO-PID), PID, Bacteria-Foraging Optimization PID (BFO-PID) etc makes ABO-PID a good addition to solving PID Controller tuning problems using metaheuristics.

## 1. Introduction

All over the world, process control is a major consideration in design and production engineering [1]. The main purpose of control systems is to steer the system in such a way as to obtain the expected dynamic response as well as the static requirements of a closed loop system [2]. In every industrial process, production engineers are concerned with ensuring that the actual output matches the predetermined results. To achieve this, some level of control procedures are required. This development has led to the design of different techniques of process control. Some of these techniques are numerical, others are neural, adaptive, metaheuristic or fuzzy control processes etc [3]. Among these several techniques, the Proportional-Integral-Derivative (PID) control systems are about the most popular.

Proportional-Integral-Derivative (PID) Controller is a feedback loop control technique that is used in many scientific, engineering and industrial control establishments, especially for systems that have been designed with accurate mathematical models [4, 5]. The PID controller calculates the three system coefficients: proportional, integral and derivative parameter values. The proportional component of the PID is concerned with the calculation of the values of the recent error; the integral component determines the output of the sum of the recent errors while the derivative component calculates the reaction of the power-generating system as a result of the rate at which the errors are changing. The calculated sum of these three activities is forwarded to the control system.

The main consideration in the use of PID controllers is to ensure the efficient and effective tuning of parameters. This is because, in practical situations, control systems have some characteristics, for example, nonlinearity, time-variability and system’s delays. Also there are situations when the systems’ parameters can change with time and operating environments [6]. Thus, the overriding objective of tuning PID controllers is to accurately obtain the parameters that appropriately satisfies the performance specifications of a closed-loop system in different operating environments. The popularity of the PID as a control procedure stems from its ease of use, simplicity of operation, ease of maintenance, low cost, ease of implementation, dynamism, effectiveness and efficiency [7]. Until the past two decades, the most popular PID has been the Ziegler-Nichols PID and the Cohen Coon PID [8].

However, the biggest problem in using PID, in general, and manual-based PID, in particular, is with the tuning of its parameters. Appropriate tuning of manual PID parameters requires technical expertise. The traditional tuning methods like the Ziegler-Nichols PID and the Cohen-Coon PID demand that the process models be minimized when they appear complex [9]. Through a procedure referred to as complex process minimization, these traditional methods, especially the Ziegler Nichols technique demands that the Integral and Derivative coefficients be set initially to zero, then the Proportional coefficient is then increased from zero unitil it gets to the predetermined Ultimate gain (*KuK*_*u*_). It is belived that at this point, the output of the control system has reached a stable level and so the oscilliations has got to a consistent level such that the oscilliation time (dead time), *P*_*u*_, is then used to set the PID coefficients [10]. Failure to adequately address this concern results in inappropriate tuning that leads to system overshoot, system-delays, steady-state errors and eventually affects the system stability [11].

The shortcomings of the traditional tuning methods like the Ziegler-Nichols PID and the Cohen-Coon PID necesitates the need for metaheuristic tuning algorithms like Genetic Algorithm PID (GA-PID) [12], Particle-Swarm Optimization PID (PSO-PID) [13], Ant Colony Optimization PID (ACO-PID) [14], PID-Tuner [15], Bacteria-Foraging Optimization PID (BFO-PID) [16] and now the African Buffalo Optimization (ABO-PID). In spite of the noble contributions of the existing metaheuristic tuning techniques, there still exists cases of systems overshoot, steady state error as well as delay in rise time and settling time [17, 18]. The need for further improvements, therefore, is the motivation for this study

The rest of this paper is organised in the following way: section two discusses the Automatic Voltage Regulators; section three examines the African Buffalo Optimization (ABO); section four focuses on the application of the ABO-PID and other techniques in tuning the parameters of a PID for an effective AVR. Moreover, section five draws conclusions on the study. This is followed by the acknowledgement of support for the study and then the references.

## 2. The automatic voltage regulators

The Automatic Voltage Regulator (AVR) is a vital component of any power generating system because helps to control the terminal voltage since it regulates the exciter voltage of the power generating system. The AVR is designed to constantly monitor the power system’s terminal voltage at all times and under any load conditions. The AVR does this by ensuring that the generator’s voltage operates within the preset system’s limits. The AVR system is composed of four basic components: amplifier, exciter, generator and sensor. The function of these AVR components can be can be represented linearly and represented mathematically as:

$$Amplifier\;\;\frac{V_{ref}\left(s\right)}{V_{e}\left(s\right)}=\frac{G_{A}}{1+\tau_{A}\left(s\right)}$$ (1)

Where *G*_*A*_ denotes the Amplifier gain, *V*_*ref*_(*s*) represents the reference voltage, *V*_*e*_ is the error voltage, and *τ*_*A*_ denotes the Time constant in the *S* domain.

The usual values of *G*_*A*_ are between 10 and 400. The time constant of an amplifier time ranges from 0.02 to 0.1 s.

The transfer function of an exciter could be represented by a gain *G*_*E*_ coupled with a single time constant

$$Exciter\;\frac{V_{F}\left(s\right)}{V_{ref}\left(s\right)}=\frac{G_{E}}{1+\tau_{E}\left(s\right)}$$ (2)

Here, *G*_*E*_ represents Exciter gain and *τ*_*E*_(*s*) is Time constant in the S domain. The usual values of *G*_*E*_ are between 10 and 400. The time constant of an Exciter is between 0.5 and 1.0 s.

Similarly, the transfer function of the generator terminal voltage in relation to its field voltage can be represented by a gain *G*_*G*_ coupled with a time constant

$$Generator\;\frac{V_{T}\left(s\right)}{V_{ref}\left(s\right)}=\frac{G_{G}}{1+\tau_{G}\left(s\right)}$$ (3)

Please note that these constants are load-dependent, *G*_*G*_ could vary from 0.7 to 1.0, and between 1.0 s to 2.0 s (from full load to no load).

Finally, the sensor could be modelled by a simple first-order transfer function:

$$Sensor\;\frac{V_{S}\left(s\right)}{V_{T}\left(s\right)}=\frac{G_{S}}{1+\tau_{S}\left(s\right)}$$ (4)

T_R_ could be very small, usually between 0.001 and 0.06 s.

**Fig 1:**
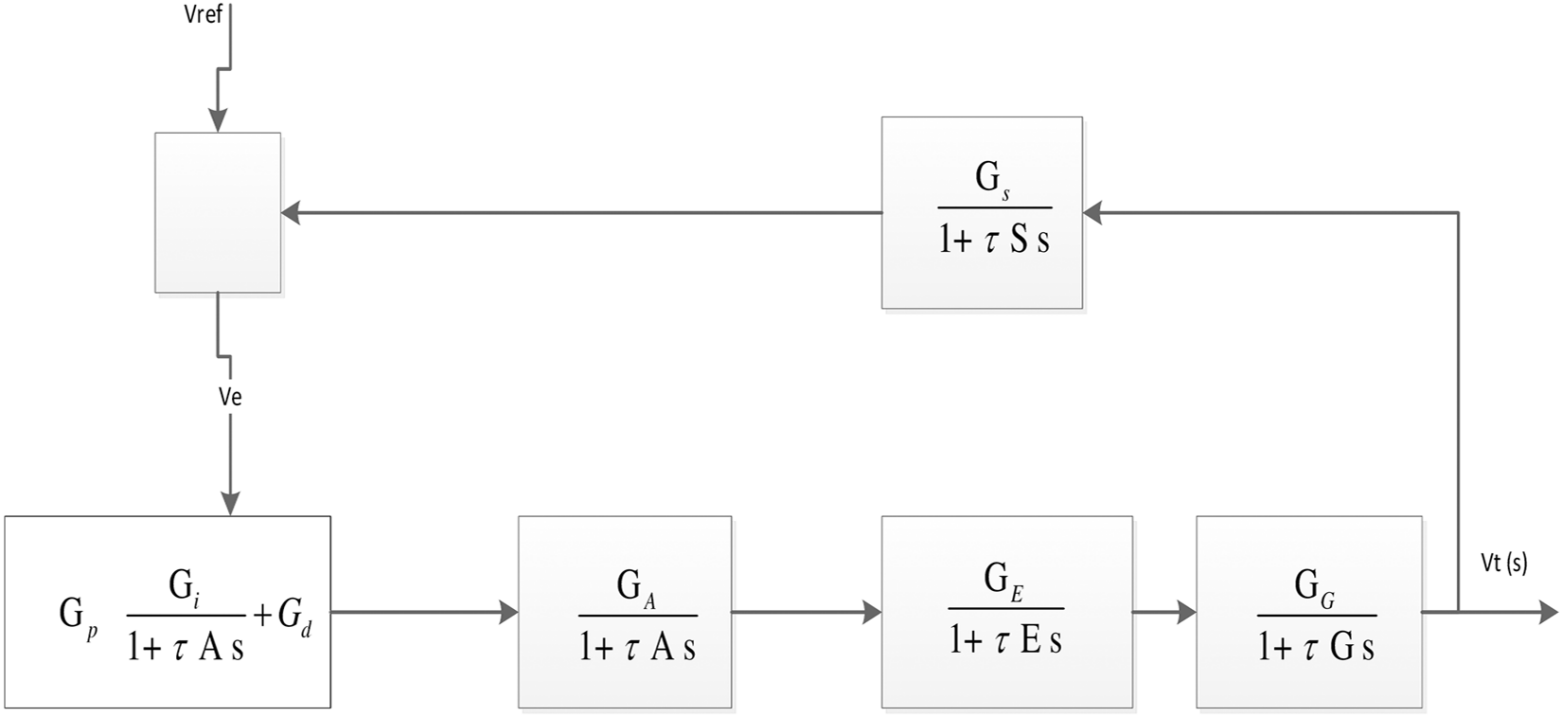
Block diagram of an AVR system with a PID.

In general, the block diagram of a PID for an AVR is as shown in Fig 1 where the Amplifier, Exciter, Generator and Sensor represent the component parts of the AVR.

Usually any remarkable increase in the generator’s reactive power load is followed by a drop in the exciter’s voltage. This underscores the need for a PID controller in an AVR so as to minimize or, possibly, eradicate the error in voltage output and achieve voltage stability. Therefore, a properly functioning AVR intrinsically controlled by a properly-tuned PID influences the voltage level by a steady-state process and reduces or even totally eliminates the voltage oscillations during fleeting periods.

## 3. The African buffalo optimization algorithm

The African Buffalo Optimization is a recently developed algorithm with deliberate application of the lean metaheuristics design principles in mind [19, 20]. One of the current research directions in metaheuristics algorithm development is the need for lean metaheuristics algorithm design totally avoiding the Frankenstein phenomena [21]. Frankenstein phenomena refers to a situation in algorithm development where designers use several parameters to the extent that the individual contributions of a particular parameter to the overall working of the algorithm becomes difficult to pinpoint [22]. Since its development, the ABO has been successfully applied to solve numerical function optimization problems [23], symmetric Travelling Salesman’s Problems [24] and the asymmetric Travelling Salesman’s Problems [25, 26].

Basically, the African Buffalo Optimization is a simulation of the movement of African buffalos from one location to the other in the vast African rainforests and savannahs in search of grazing pastures using two major vocalizations: the */waaa/* and the */maaa/* calls. The /*waaa/* call is used for exploration of the search space since where the buffalos are presently has been sufficiently grazed or unsafe for further grazing while the */maaa/* calls are used to summon the buffalos for exploitation as the grazing landscape is lush and safe. The ABO algorithm is presented below:

1 Randomly initialize the buffalos *to* nodes within the search space;
2 Update the buffalos’ exploitation fitness:
  $${\text{m}}_{\text{k}}\prime ={\text{m}}_{\text{k}}+\text{lp}1\left(bg-{\text{w}}_{\text{k}}\right)+\text{lp}2\left(bp.\text{k}-{\text{w}}_{\text{k}}\right)$$
  where m_k_ and w_k_ represents the exploitation and exploration moves respectively of the k^th^ buffalo (k = 1, 2…N); *lp*1 and *lp*2 are learning parameters; *bg* is the herd’s best fitness and *bp*, the individual buffalo’s best location.
3 Update the exploitation location of buffalos using:
  $${\text{w}}_{\text{k}}\prime =\frac{\left({\text{w}}_{\text{k}}+{\text{m}}_{\text{k}}\right)}{\lambda }$$
4 Is *bg* updating? Yes, go to 5. If No in 10 iterations, go to 1
5 If the stopping criteria is not reached, return to step 2, else go to 6
6 Output best solution.

ABO algorithm

In the ABO algorithm, w_k_ is used to represent the *waaa* (explore) calls of the buffalos with particular reference to buffalo k. Similarly, m_k_ represents the *maaa* (exploit) call, w_k_′ represents the request for further exploration; m_k_′ is a request call for further exploitation; lp1 and lp2 are the learning parameters; *λ* takes a value of 0.1 to 2 depending on the problem under investigation: the higher the value, the more the exploitation and less of exploration and vice-versa. The pseudocode of the ABO that details its step-by step implementation strategy is presented below

1 **Begin**
2  Randomly initialize the buffalos to different locations within the search space;
3   **While** (until termination), do
4    **For** k = 1: N (N = population), do
5     Evaluate the buffalos’ exploitation fitness:
6     m_k_′ = m_k_ + lpl(bg − w_k_) + lp2(bp_k_ − w_k_)
7     where m_k_ = exploitation move; w_k_ = exploration move; bg = position of the best
8     buffalo; lp1 and lp2 denotes learning parameters; bg is the
9     herd’s best fitness and bp_k_, individual buffalo’s best fitness
10     Update the location of buffalos using the Equation:
11     ${\text{w}}_{\text{k}}\prime \;=\frac{\left({\text{w}}_{\text{k}}+{\text{ m}}_{\text{k}}\right)}{\text{λ}}$
12     Is *bg* updating? Yes, go to 13. If No in 10 iterations, go to 2
13    **End for**
14   **End while**
15  Output best solution.
16 **End**

ABO pseudocode

**Fig 2:**
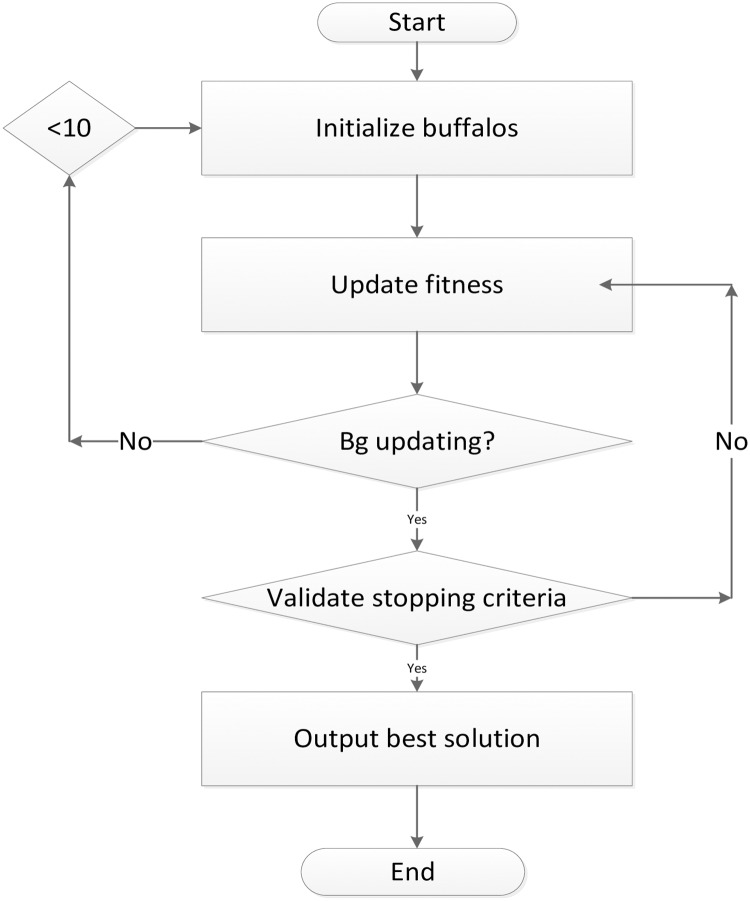
ABO flowchart.

The ABO algorithm’s flowchart in Fig 2

## 4. ABO for proportional integral derivative tuning of automatic voltage regulator

As earlier stated, the main function of a PID controller is to stabilise the dynamic response of the AVR in addition to reducing or totally eliminate the steady-state error. The PID has three major parts, namely, the Proportional, Integral and Derivative mechanisms. The Proportional component (*G*_*p*_) of a PID controller system is used to minimize the rise time of the power system. It is, however, incapable of completely eliminating the steady-state error. The primary function of the integral component (*G*_*i*_) is to reduce or possibly eliminate the steady-state error for a step input. This component is also useful in slowing down the transient response of the power system. Similarly, the derivative control (*G*_*d*_) is useful in increasing the system stability by deliberately reducing or eliminating the system overshoot, thus, enhancing the transient response of the system.

### 4.1 ABO-PID search technique

The searching mechanism of the ABO-PID controller is itemized below.

Step 1: Initialize the buffalos on the search space in sets of three buffalos per set. That is to say that if there are a population of N buffalos, they will consist of *N/3* components. Set *s* which represents the step function as 2
Step 2: Determine the exploitation fitness of each buffalo in the population using the Eqs 5 and 6 respectively
  $${\text{m}}_{\text{k}}\prime ={\text{m}}_{\text{k}}+\text{lp}1\left(bg-{\text{w}}_{\text{k}}\right)+\text{lp}2\left(bp.\text{k}-{\text{w}}_{\text{k}}\right)$$ (5)
  $${\text{w}}_{\text{k}}\prime =\frac{\left({\text{w}}_{\text{k}}+{\text{m}}_{\text{k}}\right)}{\lambda }$$ (6)
Step 3: Determine *Gp Gi and Gd* for each set of buffalos
Step 4: Plot the *Gp Gi and Gd* into the PID transfer function represented by Equation
  $$Gp\;\left(s\right)+\frac{Ki}{S}+Gd\;\left(s\right)$$ (7)
  Determine the buffalo set with the best performance and set as *bg*
Step 5: Set the values of *x/y*. If the output is 1 which represents the steady state, proceed to Step 6, else return to Step 2
Step 6: Plot the output into a MATLAB tool to determine the rise time, settling time, percentage overshoot and the steady state error.

### 4.2 PID to AVR implementations

**Fig 3:**
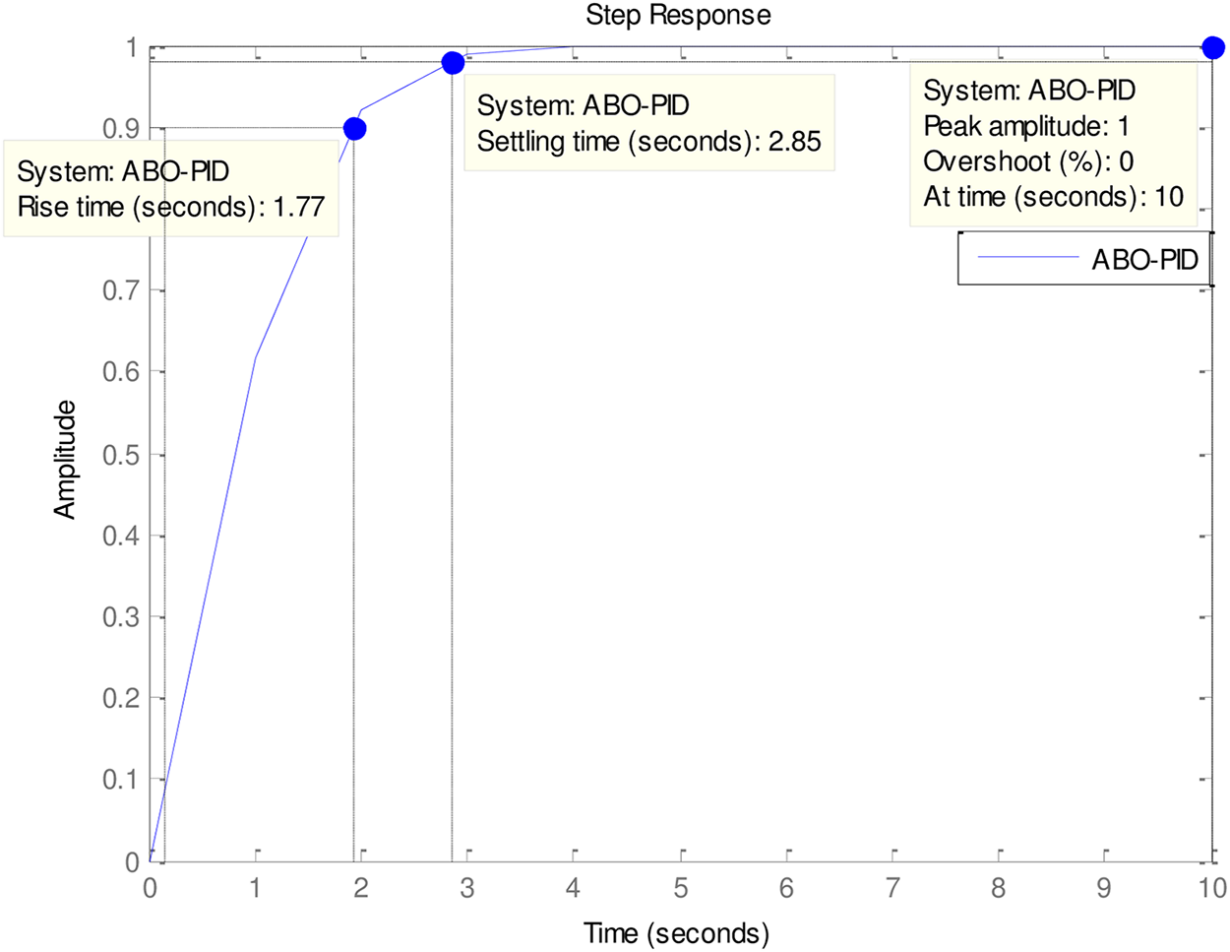
ABO-PID.

**Fig 4:**
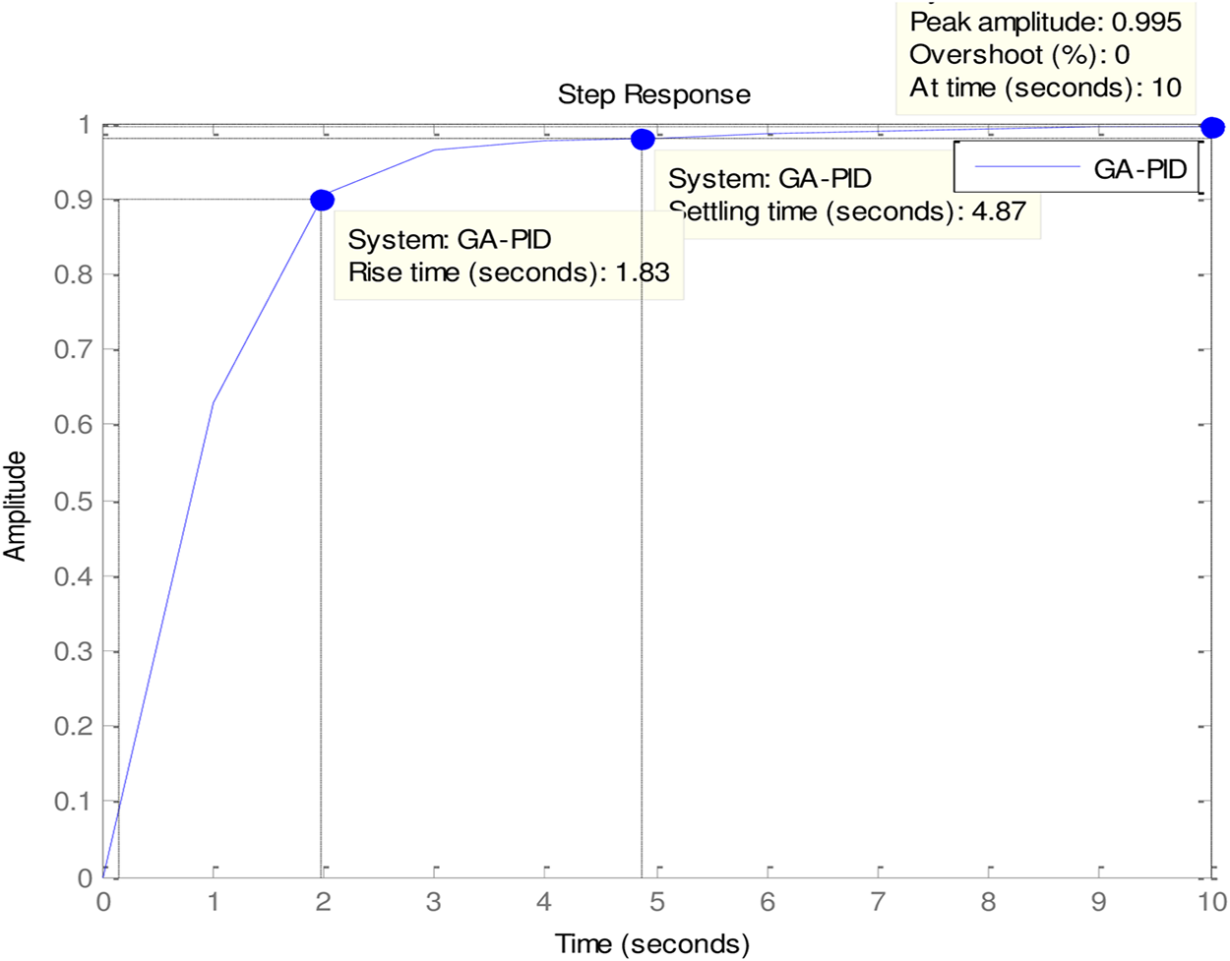
GA-PID.

**Fig 5:**
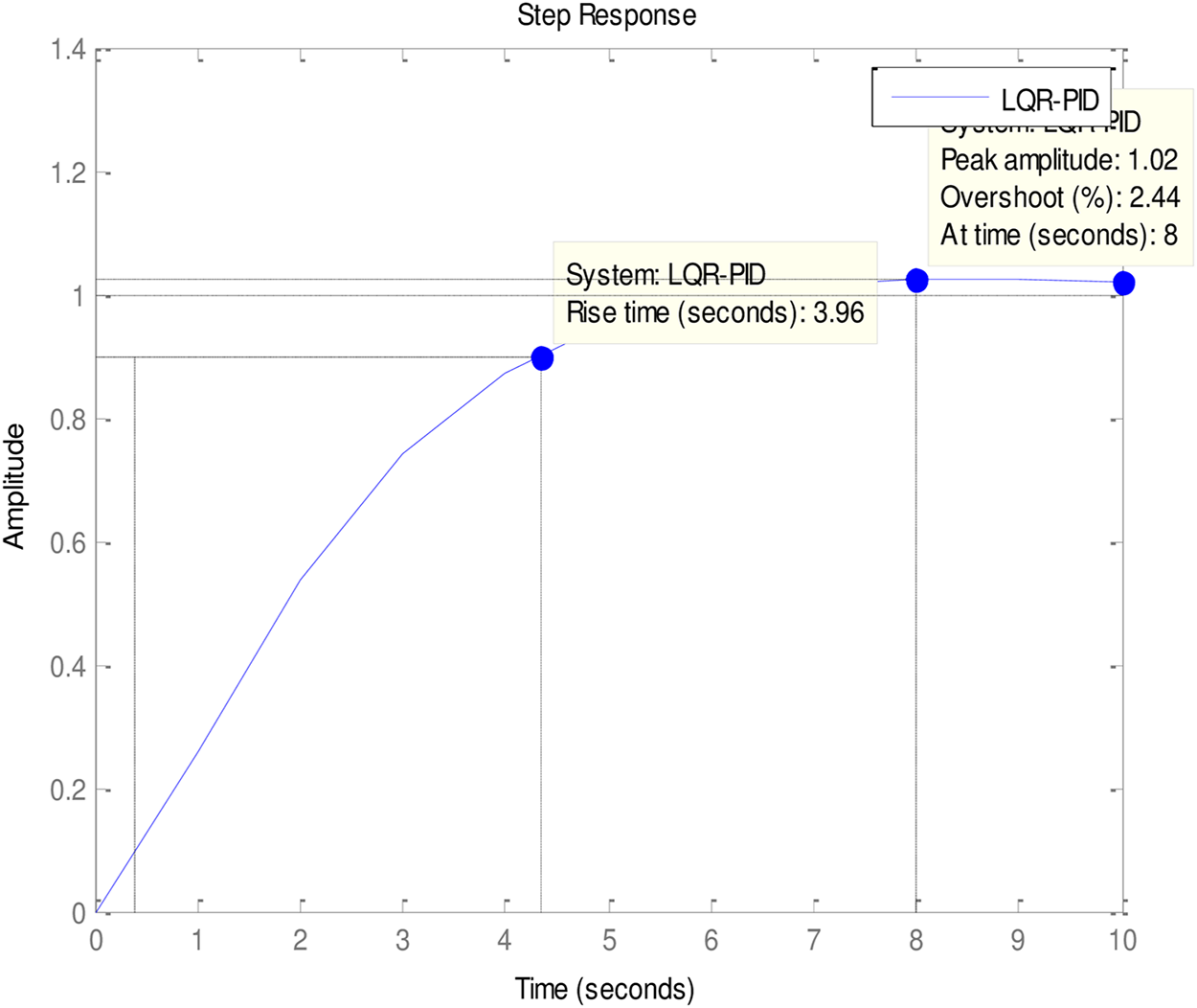
LQR-PID.

**Fig 6:**
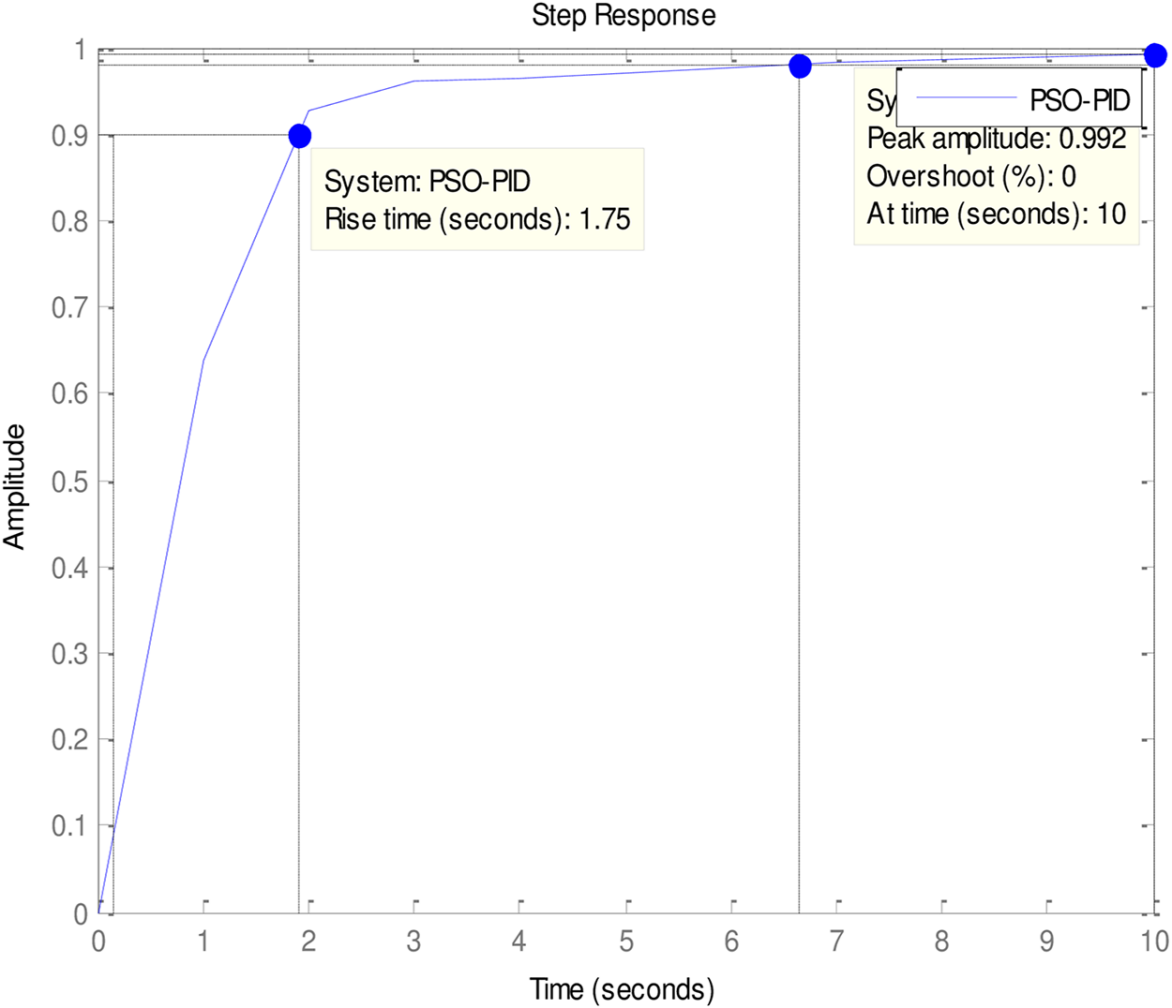
PSO-PID.

**Fig 7:**
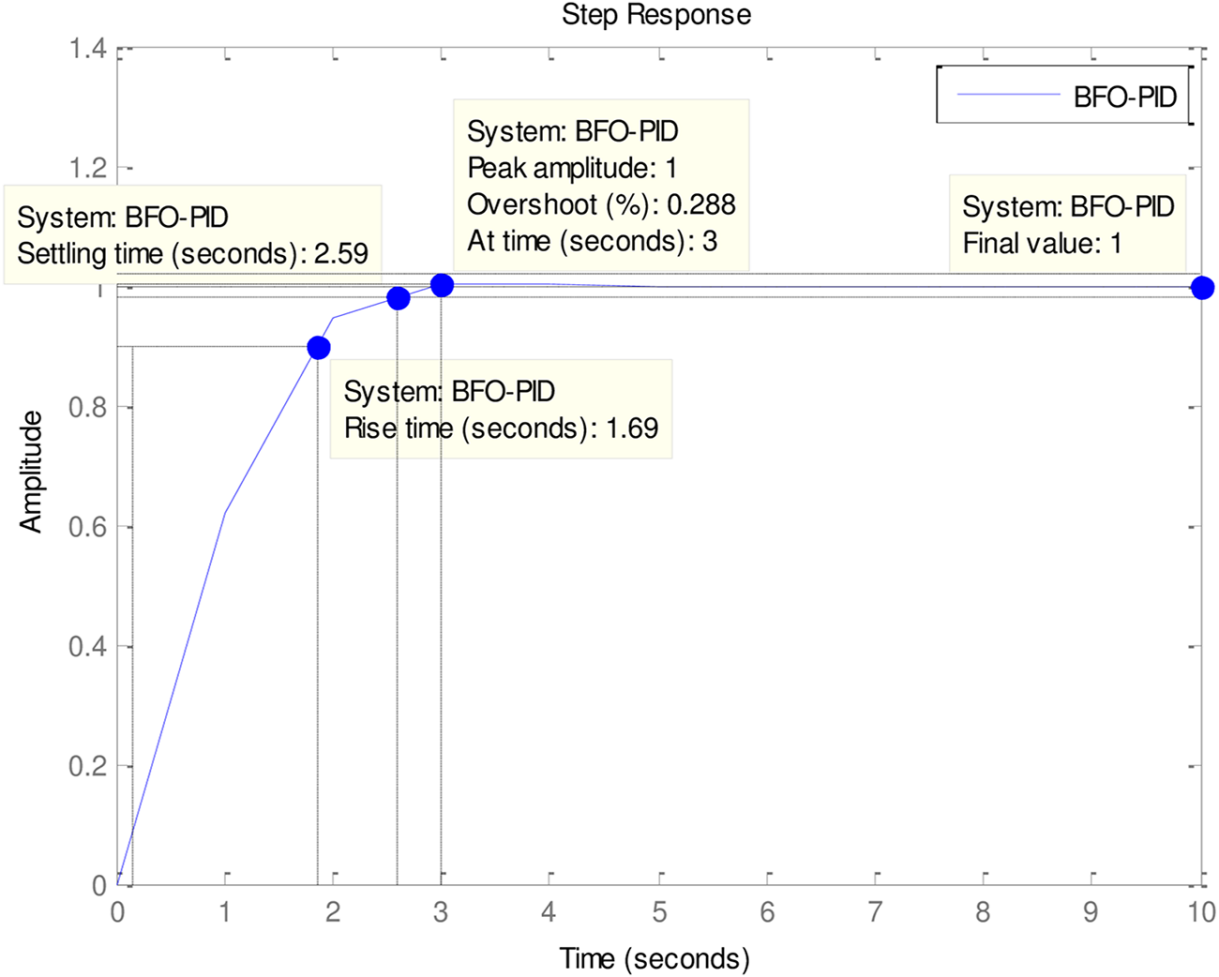
BFO-PID.

**Fig 8:**
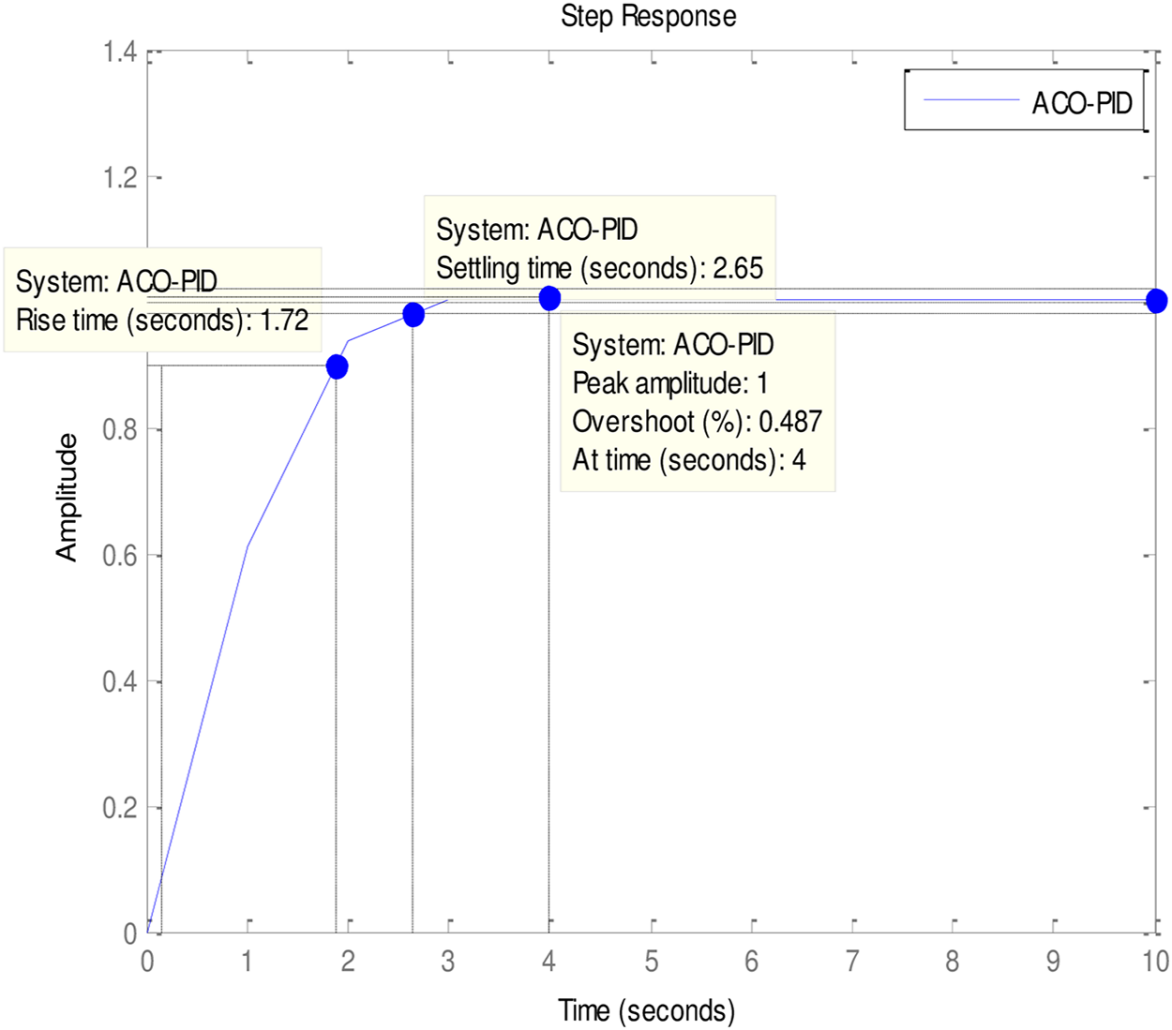
ACO-PID.

**Fig 9:**
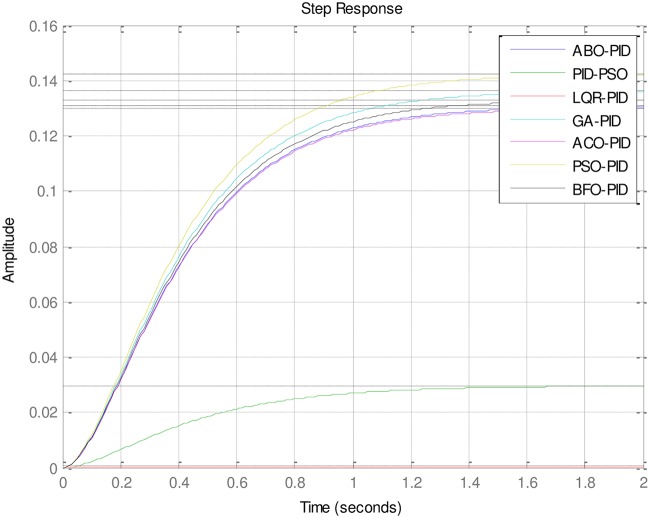
Dynamic comparative output.

An implementation of the ABO-PID, PSO-PID, ACO-PID, PSO-PID, PID-PSO, GA-PID, GA-PID, LQR-PID and BFO-PID were executed in order to test their capacity to tune the AVR (Please see Figs 3–9). The parameters are presented in Table 1:

**Table 1:** Experimental parameters.

*Parameters*	*Values*
***K*_*A*_**	10
***K*_*E*_**	1.0
***K*_*G*_**	1.0
***K*_*S*_**	1.0
***τ*_*A*_**	0.1
***τ*_*E*_**	0.4
***τ*_*G*_**	1.0
***τ*_*S*_**	0.01

It should be noted that the variable *Ve* in the Block diagram (Fig 1) is obtained by Eq 8

Ve=Vt(s)−Vref (s) (8)

In the above Equation represents, *Ve* represents the tracking error and it is obtained by subtracting the reference (input) signal (*Vref* (*s*)) from the output signal (*Vt*(*s*)). The error signal (*Ve*) is then sent to the PID controller whose duty it is to calculate the proportional, the integral and the derivative of this error signal.

Similarly, the transfer function of a PID Controller is:

$$Gp+\frac{Ki}{S}+Gd\;\left(s\right)$$ (9)

Also, the transfer function of the AVR components is:

$$\frac{\Delta Vt\;\left(s\right)}{\Delta Vref\;\left(s\right)}=\frac{\left(S^{2}\;Gd+S\;Gp+Gi\right)\;\left(K_{A}\;\;K_{E}\;\;K_{G\;\;K_{S}}\right)\;\left(1+S\;T_{S}\right)}{S\left(1+S\;\tau_{A}\right)\;\left(1+S\;\tau_{E}\right)\;\left(1+S\;\tau_{G}\right)\;\left(1+\;S\tau_{S}\right)\;\left(S^{2}\;Gd+S\;Gp+Gi\right)\;\left(K_{A}\;\;K_{E}\;\;K_{G\;\;K_{S}}\right)}$$ (10)

The ABO-PID was implemented in a MATLAB code, executed using a MATLAB 2012b complier. The outcome of this experiment is compared with results from other optimization algorithms such as PSO-PID, ACO-PID, PSO-PID, PID-PSO, GA-PID, LQR-PID, and BFO-PID (See Figs 3–9). The simulation result is presented in Table 2.

**Table 2:** Simulation results.

Gain Overshoot (%)	Type of controller	PID Parameters			Rise Time (secs)	Settling Time (secs)	Steady State Error
		*G*_*p*_	*G*_*i*_	*G*_*d*_			
0	ABO-PID	3.007	1.0734	0.4304	1.77	2.85	0
8.99	PID_PSO	0.6125	0.4197	0.2013	0.684	3.087	0.06
2.44	LQR-PID	1.0100	0.5000	0.1000	0.500	2.335	0.02
0	GA-PID	3.1563	0.9463	0.4930	0.493	8.900	0.005
0.487	ACO-PID	2.9917	1.1053	0.3085	0.493	7.100	0
0	PSO-PID	3.3172	0.8993	0.2814	0.4993	10.200	0.008
0.288	BFO-PID	3.0725	1.1054	0.2601	0.4993	6.800	0

Table 2 presents an interesting fact that authenticates the No Free Lunch theorem of optimization algorithms [27]. This Table highlights the strengths and weaknesses of each of the comparative algorithms to the extent that a parameter that is of particular interest to a researcher will determine his choice of an algorithm. It must be observed, nonetheless, that the ABO had a remarkable run in this set of experiments since it was the only Tuner that had 0% gain overshoot as well as 0% steady state error. Other good performers in these counts (gain overshoot and steady state error) are the GA-PID with 0% gain overshoot and 0.005% steady state error and PSO-PID with 0% gain overshoot and 0.008% steady state error. The other Tuners were not as good in these counts.

In terms of the Rise time, however, the ABO-PID had its worst result (1.77 seconds). The GA-PID and the ACO-PID were the fastest with 0.493 seconds, followed by the BFO-PID and the PSO-PID with 0.49993 seconds, LQR-PID with 0.500 seconds and PID-PSO with 0.684 seconds. With respect to the Settling time, the LQR-PID proved to be the best with 2.3355 seconds, closely followed by the ABO-PID with 2.85 seconds and PID-PSO with 3.087 seconds. The other metaheuristic tuners were rather slow in their settling times. The ABO rise time was rather slow because of the algorithm’s search procedure that requires the calculation of both exploitation (/maaa/) fitness and exploration (waaa) fitness of each buffalo in each iteration before converging at a solution. The algorithm achieves relatively fast convergence because of its use of just two controlling parameters, the lp1 and lp2. The use of few parameters results in fewer evaluations per iteration since an algorithm is required to evaluate each of its parameters before arriving at a solution.

Of special interest is the performance of FO-PSO-PID which had no gain overshoot but has the slowest rise time and setting time. That is to say that it sacrificed the rise time and settling time in its attempt to have 0% gain overshoot (See Fig 7).

### 4.3. More experimental evaluations

**Fig 10:**
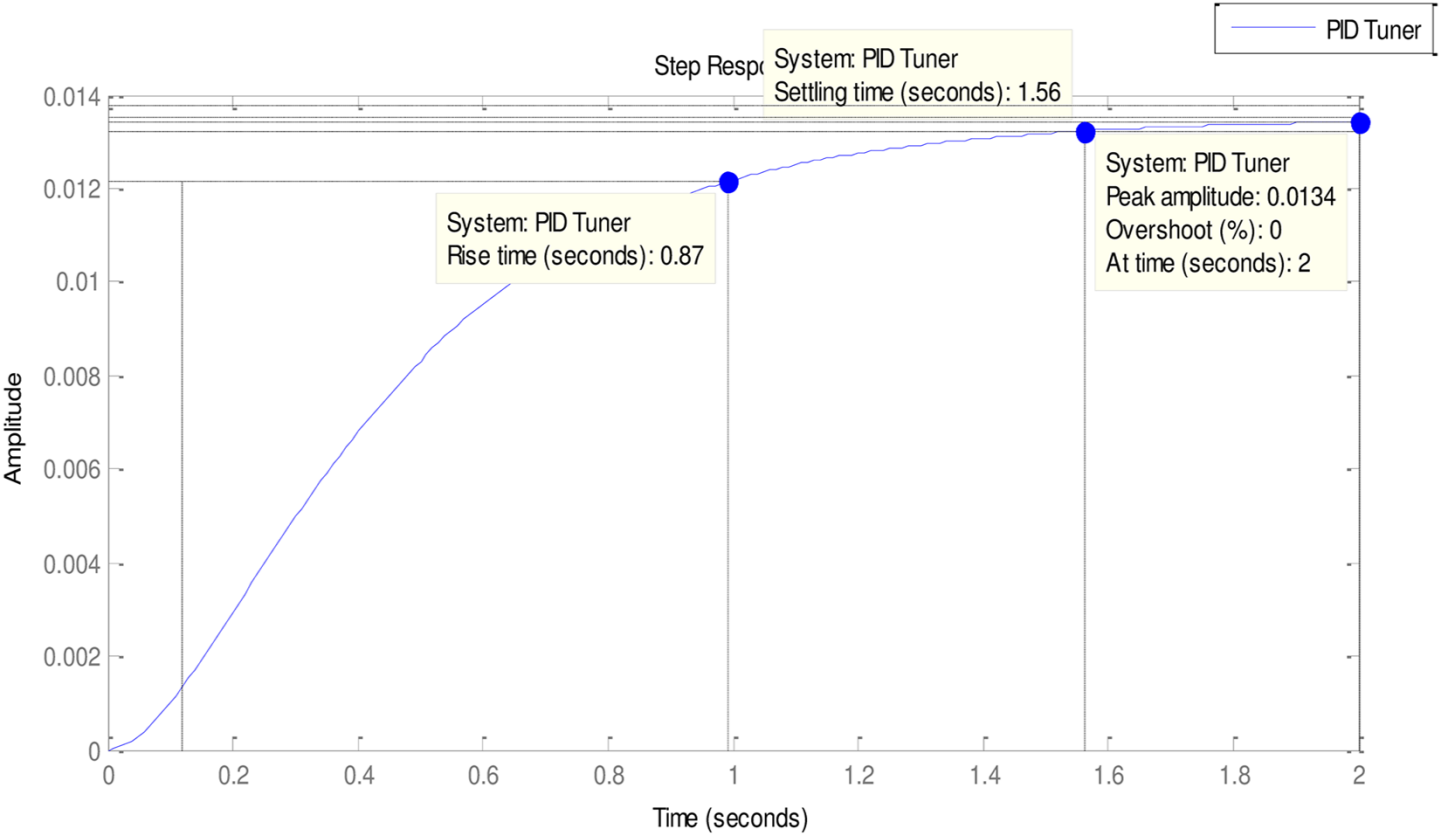
PID tuner.

**Fig 11:**
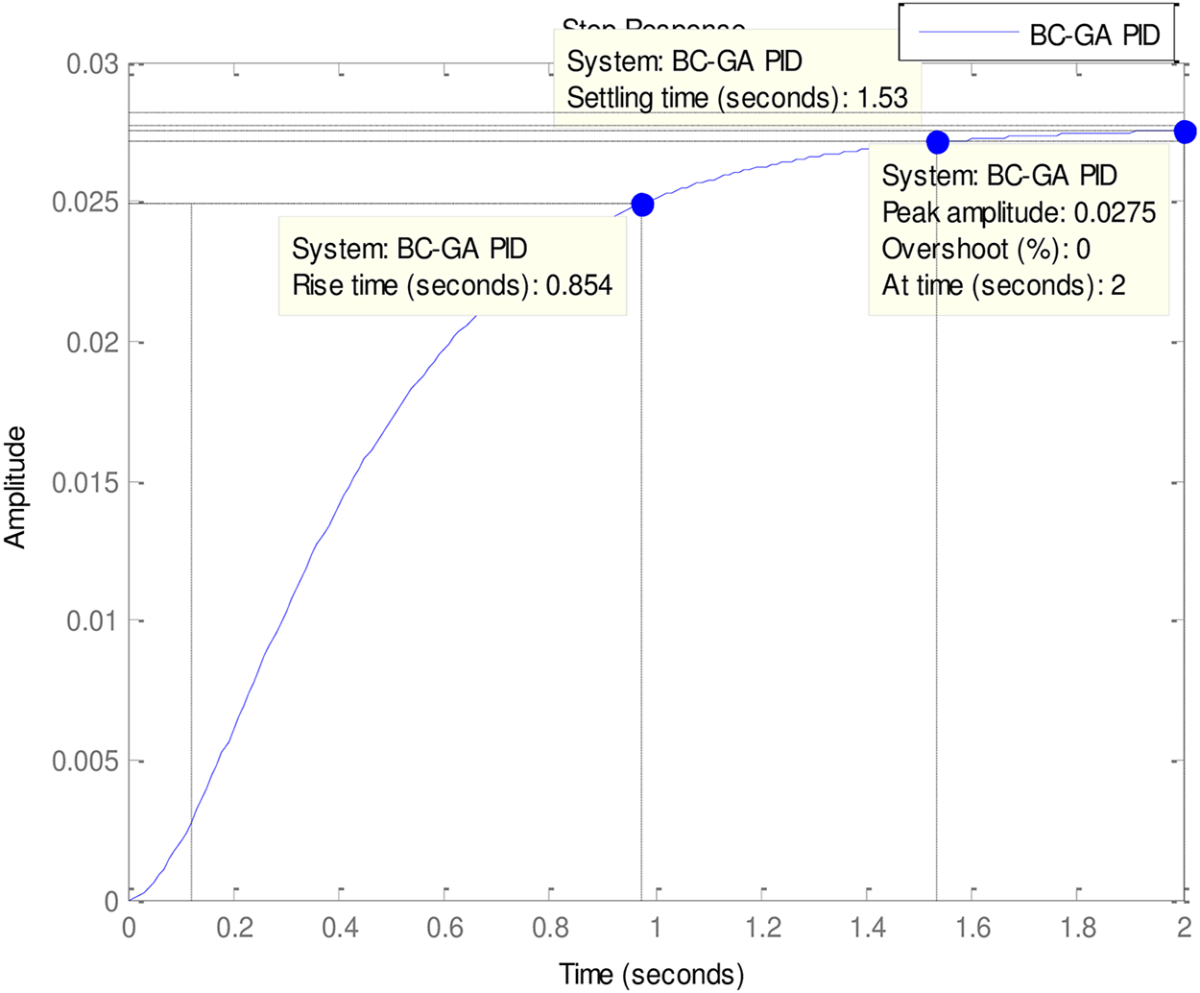
BC-GA-PID.

**Fig 12:**
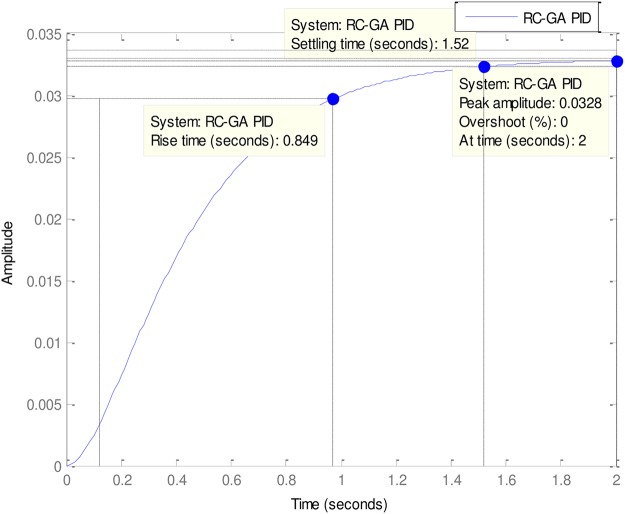
RC-GA-PID.

**Fig 13:**
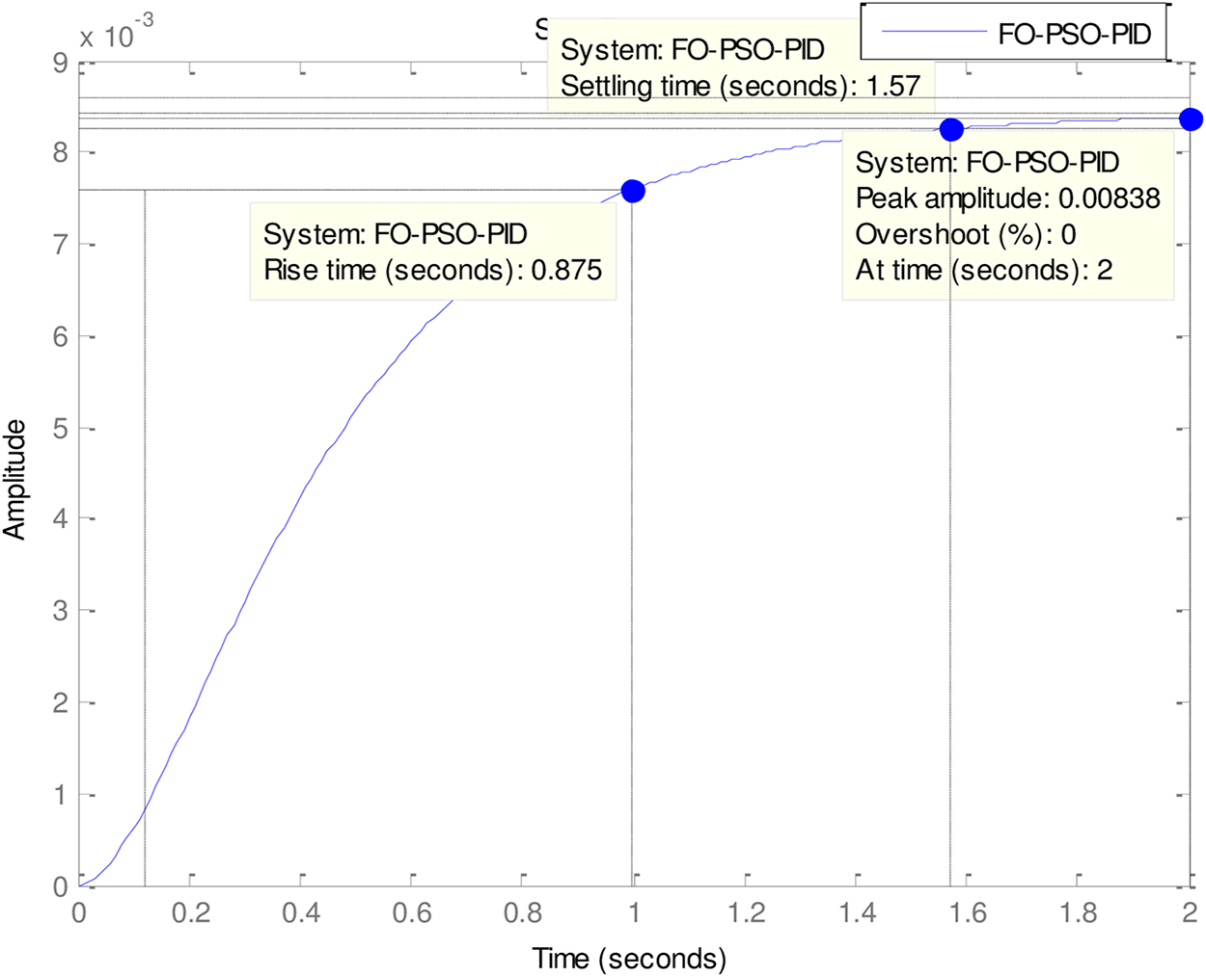
FO-PSO-PID.

In the light of the good of the good performances of the Tuners in Table 2, it is necessary to examine the performances of some other less popular but very effective Tuners vis-à-vis the ABO’s performance. These other Tuners are mostly hybrid Tuners since they are designed from some hybridized algorithms. The comparative Tuners here are the PID-Tuner [28], Real Coded Genetic Algorithm PID (RC-GA PID) and the Binary-Coded Genetic Algorithm (BC-GA PID) PID [29] (See Figs 10–13).

**Fig 14:**
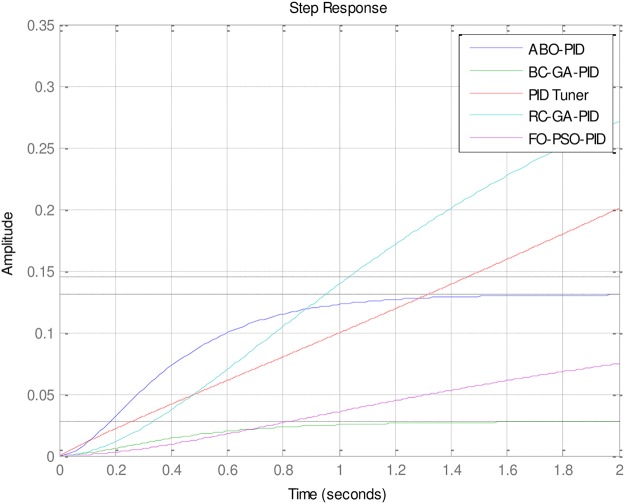
Dynamic performance output.

As can be observed in Table 3, all the Tuners here performed extremely well. In fact, all the Tuners obtained 0% gain overshoot (Please see Figs 10–13). Nevertheless, the ABO-PID was the only Tuner able to obtain 0% steady state error in addition to 0% gain overshoot. Moreover, in terms of the Rise time, the fastest Tuner in Table 3 is RC-GA PID with 0.849 seconds, followed closely by BC-GA PID with 0.0.854 seconds and the PID-Tuner with 0.87 seconds. Just like in Table 2, the ABO-PID is neither a fast riser nor a fast settler. It had the slowest Rise time and Settling Time here. Aside having the fastest Rise time, the RC-GA also had the fastest Settling time of 1.52 seconds, followed by the BC-GA with 1.53 seconds and the PID-Tuner with 0.56 seconds (See Fig 14).

**Table 3:** More experiemental results.

Gain Overshoot (%)	Type of controller	PID Parameters			Rise Time secs)	Settling Time (secs)	Steady State Error
		*G*_*p*_	*G*_*i*_	*G*_*d*_			
0	PID_TUNER	0.2736	0.1723	0.1150	0.87	1.56	0.0134
0	ABO-PID	3.007	1.0734	0.4304	1.77	2.85	0
0	BC-GA	0.5692	0.2484	0.1258	0.854	1.53	0.028
0	RC-GA	0.6820	0.2660	0.1790	0.849	1.52	0.033
0	FO-PSO-PID	0.1700	0.0300	0.0140	0.875	1.57	0.008

In the light of the performances of the comparative Tuners in Table 3, a trend could be noticeable and that is that the Tuners Rise times correlates with Settling times. That is to say, that the first Tuner to rise will also, likely, be the first to settle and vice-versa (refer to Figs 10–13). From the foregoing analysis, it can be adduced that a good tuner is one that does a fair trade off in balancing the performance of different parameters in its quest to obtain effective and efficient tuning. The strength of the ABO-PID stands out in its ability to maintain a good balancing of the power generating parameters, thus ensuring harmonious, effective and efficient working of the AVR system.

## 5. Conclusion

The sudden but steady popularity of Proportional-Integral-Derivate (PID) controllers to the tuning of vital paramters in power engineering devices such as DC motors and Automatic Voltage Regulators is attracting the attention of researchers. This popularity stems from their simple implementation strategies, robustness, efficiency and effectiveness of PID controllers. The main consideration in designing PID controllers is ensuring the appropriate tuning of its parameters. Metaheuristic tuning of PID controllers has proven to be much more versatile, efficient and effective than manual tuning which is rather difficult and time-consuming. This success of metaheuristic tuning of PID paramaters has led to the application of metaheuristics like the Genetic Algorithm (GA), Ant Colony Optimization (ACO), Particle Swarm Optimization (PSO) and the Fruit Fly Optimization Algorithm (FOA) etc. to PID parameters-tuning.

In the light of the above, this paper applies the African Buffalo Optimization to tune the PID Controller’s parameters of the AVR with the aim of improving the results from the existing metaheuristic tuning techniques. After a number of experimental evaluations, the ABO-PID has been proven to be quite effective. Comparative experimental results indicate that the ABO-PID was the only method that obtained optimal solution (0%) in the gain overshoot and steady steady error indices. Next to the ABO-PID was the GA-PID that obtained 0% in gain overshoot and 0.005% in the steady state error. Also the performance of the PSO-PID was very commendable with 0% gain overshoot and 0.008% steady steady state error. The ACO-PID obtained 0.487% gain overshoot and 0% steady state error which is also a good performance. However, it must be observed that the ABO-PID needs further improvement in its Rise time and Settling time.

From the foregoing discussion, therefore, it is safe to conclude that the newly-designed ABO-PID controller has proven to be a reliable algorithm for the parameter tuning of AVR. The ABO-PID controller has displayed superior tuning capability of PID parameters of an AVR system by achieving a good trade-off between the time-domain indices in arriving at a stable power system. However, in tandem with the No Free Lunch theorem [27], it is recommended that the parameter of utmost relevance to a researcher may determine his choice of a metaheuristic Tuner. If the most important consideration is the 0% gain overshoot and 0% steady state error, then the ABO-PID is the obvious choice. But if a researcher is more concerned with a system that has the fastest rise time the GA-PID and the ACO-PID are better choices. Similarly, if a system with the fastest settling time is the primary concern, then the RC-GA PID and BC-GA PID are the obvious choices. Finally, we recommend further experimental investigations of other metaheuristic tuners of AVR not covered in this study for the benefit of researchers and practitioners in search of efficient and effective Tuners.
